# Summary of the National Advisory Committee on Immunization (NACI) Statement: Updated guidance on respiratory syncytial virus (RSV) vaccines for older adults and for adults at high risk of severe RSV disease

**DOI:** 10.14745/ccdr.v52i06a02

**Published:** 2026-06-30

**Authors:** Joseline Zafack, Elissa M Abrams, Nicholas Brousseau

**Affiliations:** 1 Centre for Immunization Surveillance and Programs, Public Health Agency of Canada, Ottawa, ON; 2 Department of Pediatrics and Child Health, Section of Allergy and Immunology, University of Manitoba, Winnipeg, MB; 3 Department of Pediatrics, Division of Allergy, University of British Columbia, Vancouver, BC; 4 Département de médecine sociale et préventive, Faculté de médecine, Université Laval, Québec, QC

**Keywords:** National Advisory Committee on Immunization, respiratory syncytial virus, RSV, adults, RSVPreF3/Arexvy, RSVpreF/Abrysvo, mRNA-1345/mRESVIA, vaccine

## Abstract

**Background:**

Respiratory syncytial virus (RSV) is a common respiratory virus that can have serious complications for older adults and adults with certain medical conditions. Since July 2024, the National Advisory Committee on Immunization (NACI) has provided immunization guidance for the protection of older adults against RSV. In April 2026, NACI updated its guidance to reflect emerging epidemiological data identifying younger adults at high risk of severe RSV and evidence on the benefits of RSV vaccines in older adults and adults at high risk.

**Methods:**

NACI reviewed available evidence on the benefits and risks of RSV vaccination in older adults and individuals at higher risk, as well as additional factors, including ethics, equity, feasibility, acceptability and cost-effectiveness.

**Results:**

The RSV disease burden in younger adults with certain underlying conditions is comparable to that in older adults. Among older adults, RSV infection may increase the risk of cardiovascular events and Guillain-Barré syndrome (GBS). The RSV vaccines demonstrate strong protection against RSV-associated lower respiratory tract disease and severe outcomes, with favourable safety profiles. Monitoring for rare adverse events following immunization, total duration of protection, and need for boosters is ongoing.

**Conclusion:**

NACI expanded the use of RSV vaccines to some adults at high risk for whom the balance of clinical benefits, safety and feasibility is favourable. NACI strongly recommends RSV immunization programs for all adults aged 75 years and older; adults aged 65–74 who are at increased risk of severe RSV disease; and adults aged 18 years and older who reside in chronic care facilities, have received a lung or hematopoietic stem cell transplant, are receiving home or chronic oxygen therapy, or are receiving dialysis. For other adults, vaccination may be considered on an individual basis.

## Introduction

Respiratory syncytial virus (RSV) is a common respiratory virus that can be associated with serious complications, including hospitalization, intensive care unit admission, and death, among older adults and adults with certain medical conditions (1–3). Risk for severe outcomes increases with age, residence in chronic care or long-term facilities, presence of comorbidities (e.g., cardiorespiratory disease, metabolic disease, immunocompromise, chronic liver or kidney disease, neurologic disease, or class 3 obesity), or due to factors related to the social determinants of health (1). Recent data suggest a higher burden of RSV-associated hospitalization than previously reported (2,3). Among older adults, RSV infection may increase the risk of Guillain-Barré syndrome (GBS) and cardiovascular events, such as ischemic heart disease, stroke, or heart failure (3,4).

There are three vaccines available to protect older adults and adults at high risk of severe disease from RSV in Canada: RSVPreF^3^ (AREXVY, GSK), which is authorized to protect individuals 60 years of age and older, and individuals 50–59 years of age at increased risk of RSV disease; and RSVpreF (ABRYSVO®, Pfizer) and mRNA-1345 (mRESVIA®, Moderna), which are authorized to protect individuals 60 years of age and older, and individuals 18–59 years at increased risk of lower respiratory tract disease caused by RSV.

## Methods

Evidence synthesis was performed by the National Advisory Committee on Immunization (NACI) Secretariat and reviewed by the NACI RSV Working Group. This included such considerations as the burden of RSV disease in target populations; safety, immunogenicity, efficacy, and effectiveness of the vaccines; and other aspects of the overall immunization strategy (ethics, equity, feasibility, acceptability and cost-effectiveness) (3,5). The evidence and programmatic considerations were organized by the NACI Secretariat using a process informed by the Grading of Recommendations, Assessment, Development and Evaluation (GRADE) framework and all of the information was used to facilitate development of NACI guidance. Further information on NACI’s evidence-based methods is available in *Evidence-based recommendations for immunization–Methods of the National Advisory Committee on Immunization* (6).

Recommendations were developed through discussion of the RSV Working Group, then reviewed and approved by the full NACI committee.

## Results

Clinical trials and observational studies demonstrate that RSV vaccines provide meaningful protection against RSV-associated lower respiratory tract disease. NACI judged that all three vaccines work well from a clinical perspective, though there are currently less data available for the safety and efficacy/effectiveness of mRNA-1345 compared to the protein subunit vaccines (3).

All three vaccines have demonstrated favourable safety profiles. An increased risk of GBS has been identified in individuals aged 65 years and older following receipt of the RSVpreF and RSVPreF^3^ vaccines (3). At this time, it is unclear whether this increased risk will also be seen following mRNA-1345 or among adults aged less than 65 years (3). NACI will continue to monitor safety data on all RSV vaccines (RSVpreF, RSVPreF^3^, and mRNA-1345) as they become available.

Available evidence demonstrates that a single dose of RSV vaccine provides protection from RSV disease for at least three years (3). It is not yet known if the immune responses to these vaccines can be boosted with further vaccine doses. As a result, younger adults at low or moderate risk may wish to defer vaccination until a time when they are at greater risk.

Economic evidence was derived from an environmental scan of recently published economic evaluations of RSV vaccination in adult populations at increased risk of RSV and an updated Canadian cost-utility model for individuals 50 years and older. Together, these findings indicate that extending immunization programs to include some younger adults at increased risk of severe RSV disease may provide good value for money (3).

## Discussion

NACI recommendations on respiratory syncytial virus (RSV) vaccines for public health program-level decision making

The following are recommendations for provinces/territories making decisions for publicly funded immunization programs (3):

**Recommendation 1:** NACI continues to recommend that RSV immunization programs should include all adults 75 years of age and older **(*Strong NACI recommendation*)**.

**Recommendation 2:** NACI recommends that RSV immunization programs should include adults 65–74 years of age who are at increased risk of severe RSV disease **(List 1; *Strong NACI recommendation*)**.

**Recommendation 3:** NACI recommends that RSV immunization programs should include adults 18 years of age and older, who:

• Are residents of nursing homes and other chronic care facilities.

• Have had a lung transplant.

• Have had a hematopoietic stem cell transplant (in the previous two years or who remain on immunosuppression).

• Are on home oxygen or require chronic oxygen therapy regardless of living at home or elsewhere.

• Are receiving dialysis **(*Strong NACI recommendation*)**.

**List 1: Clinically significant chronic health conditions for which respiratory syncytial virus (RSV) vaccination is particularly important**

**Table:** 

• Cardiac or pulmonary disorders (includes chronic obstructive pulmonary disease, asthma, cystic fibrosis, and conditions affecting ability to clear airway secretions) • Diabetes mellitus and other metabolic diseases • Moderate and severe immunodeficiency (refer to the list of immunocompromising conditions developed for COVID-19) • Chronic renal disease • Chronic liver disease • Neurologic or neurodevelopmental conditions (includes neuromuscular, neurovascular, neurodegenerative [e.g., dementia], neurodevelopmental conditions, and seizure disorders, but excludes migraines and psychiatric conditions without neurological conditions) • Class 3 obesity (defined as BMI of 40 kg/m^2^ and over)

Abbreviation: BMI, body mass index

### Recommendations for individual-level decision making

The following recommendation is for healthcare providers advising individual clients:

**Recommendation 1:** NACI recommends that RSV vaccines may be considered for adults 18–64 years of age who are at increased risk of severe RSV disease, based on an individual decision informed by discussion with their healthcare provider **(*Discretionary NACI recommendation*)**.

## Conclusion

Respiratory syncytial virus represents a substantial source of morbidity in older adults and adults at increased risk. In this update, NACI has recommended a small expansion of the adult RSV vaccine program to also include some groups of adults with medical conditions that place them at higher risk of severe RSV disease. This update reflects recent expanded age indications authorized by Health Canada and new national and international data identifying certain medical conditions as being at higher risk of severe RSV disease.

Successful RSV programs will depend not only on efficacy, but also on feasibility, access, and equity. Jurisdictions should consider local epidemiology, resource allocation, and population needs when considering RSV vaccination strategies.
